# A variant branching pattern of the Aortic Arch: a case report

**DOI:** 10.1186/1749-8090-6-29

**Published:** 2011-03-13

**Authors:** Mange Manyama, Peter Rambau, Japhet Gilyoma, William Mahalu

**Affiliations:** 1 Bugando University College of Health Sciences, Mwanza, Tanzania; 2 Department of Surgery, Bugando Medical Centre, Mwanza, Tanzania

## Abstract

Variant aortic arch branching pattern may occur with different embryological mechanisms. We report on a variant aortic arch branching in a 41-year old Tanzanian male cadaver during dissection practice. The left common carotid artery was seen originating from the root of the brachiocephalic trunk and the left vertebral artery from the arch of the aorta proximal to the origin of the left subclavian artery. We discuss the relative literature, its potential embryologic development and clinical significance.

## Background

The aortic arch is a continuation of the ascending aorta, being located in the superior mediastinum. Three branches, the brachiocephalic trunk, left common carotid artery and left subclavian artery usually branch from the aortic arch. These branches may branch from the beginning of the arch or the upper part of the ascending aorta by varying distances between them. The brachiocephalic trunk later divides into right common carotid artery and right subclavian artery. Variations in the branching pattern of the aortic arch range from differences in the distance between origins of different branches to number of branches [1,2]. Reported variations in the aortic arch branching pattern include left common carotid artery originating from the brachiocephalic trunk; right common carotid artery and right subclavian artery originating individually from the aortic arch [2,3]. Additionally, left common carotid artery and left subclavian artery may have a common origin in the form of the left brachiocephalic trunk from the aortic arch. The left vertebral artery may also arise between the left common carotid artery and left subclavian artery [4].

In this case report, we present a variant of aortic arch branching pattern where the left common carotid artery was seen to arise from the root of the brachiocephalic and the left vertebral artery from the arch of the aorta proximal to the origin of the left subclavian artery. We discuss their embryological, clinical and surgical implications.

## Case report

**Figure 1:**
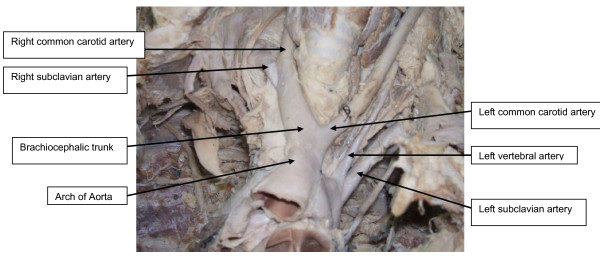
**Photograph shows the variant branching pattern of the aortic arch**.

The present report describes anomalies in branching of the aortic arch identified in a 41-year-old Tanzanian male cadaver during dissection classes for medical undergraduates in the anatomical laboratory of Bugando University College in Tanzania. From the records, he had no past medical history suggestive of cardiovascular disease. His weight and height were 75 kg and 1.70 m respectively. The cause of death was non-cardiovascular disease. The cadaver was formalin-fixed. After removal of the anterior thoracic wall, fat tissue and the pericardium covering the ascending aorta and the great vessels, we found the left common carotid artery originating from the root of the brachiocephalic trunk. This artery then crossed the trachea anteriorly from left to right and then entered the left side of the neck (Figure 1). In addition, the left vertebral artery was observed to arise from the arch of the aorta proximal to the origin of the left subclavian artery (Figure 1). The further course, branching and termination pattern of these arteries (brachiocephalic trunk, left common carotid, vertebral and left subclavian arteries) were normal. None of the laboratory or radiological investigation detected this variation prior to death.

## Discussion and Conclusions

Three classical branches spring from convex aspect of the aortic arch: the brachiocephalic trunk, left common carotid artery and left subclavian artery [1]. This branching pattern is the most common accounting for about 65% of aortic arch branching pattern. However, variation in the aortic arch branching pattern has been reported widely [2,5,6]. In analysis of 113 aortic arches in Kenya there was 67.3% of the usual pattern, the remaining 32.7% showed a great variety of patterns, the most common (25.7%) was two branches namely the left subclavian artery and a common stem that gave rise to the brachiocephalic trunk and left common carotid artery [5]. Anson's observation from 1000 aortic arches found a 65% of the usual pattern, a 25% of the four large arteries branching separately, the remaining 5% showed a great variety of patterns, the most common being symmetrical right and left brachiocephalic trunks [2]. Other variations include the left vertebral artery arising between the left common carotid and the subclavian. More rarely, the common carotid artery may be absent on one or both sides, the external and internal carotid arteries arising separately, or both carotids and one or both vertebrals may be separate branches [1,7]. The left common carotid artery varies in origin more than the right. The vertebral artery is classically described as first branch of ipsilateral subclavian artery. However, multiple variations in the origin of the vertebral artery have been reported in the literature, the most frequent variant (2.4-5.8%) is the left vertebral artery arising directly from the aortic arch between the left common carotid artery and left subclavian artery [8]. Variation in the distance between origin of these vessels has also been reported, the most frequent being approximation of the left common carotid artery to the brachiocephalic trunk.

Developmental anomalies in aortic arch branching pattern arise from unusual patterns of development of the embryonic aortic arch system of the pharyngeal arches, such that there may be persistence of aortic arches that normally disappear or disappearance of parts that normally persist [9]. Several kinds of uncommon defect occur when arches persist instead of becoming obliterated or vice versa. The proximal part of the third aortic arch normally gets extended and absorbed into the left horn of aortic sac. If it gets absorbed into the right horn of the aortic sac, it can lead to anomalies where the left common carotid artery arises from the brachiocephalic trunk. Origin of vertebral arteries from the aorta suggests that part of the aortic arch arises from the left 7th inter-segmental arteries or there was increased absorption of embryonic tissue of the left subclavian artery between origin of aortic arch and the vertebral artery [9]. These two scenarios could explain the findings in the case described in this study.

Although anomalous origins of the aortic arch branches are merely anatomic variants, knowledge of variations in the branching pattern of the aortic arch is of great importance in patients who have to undergo four-vessel angiography, aortic instrumentation, or supraaortic thoracic, head and neck surgery [10]. It has been reported that anomalies of the aortic arch branching pattern could lead to cerebral abnormalities by altering the pattern of flow in cerebral vessels [11]. In addition, knowledge of abnormal branches originating from the aortic arch is also important in the diagnosis of intracranial aneurysms following subarachnoid haemorrhage [3]. A variant of origin and course of a great vessel arising from the aortic arch is of great clinical value, because lack of knowledge of these variations may cause serious surgical complications during procedures occurring in the superior mediastinum and the root of neck.

To the best of our knowledge, this is the first case to be reported in Tanzania. Ogeng'o et al. (2010) [5] reported that over 30% of the Kenyan population may show variant branching pattern of the aortic arch. Since aortic arch anomalies may show a population variation it would be interesting to see the results of a similar study in the Tanzanian population.

## Consent

A written consent was obtained by the cadaver's next of kin for publication of the article. A copy of the written consent is available for review by the Editor-in-Chief of this journal.

## Competing interests

The authors declare that they have no competing interests.

## Authors' contributions

MM did the dissection, obtained the photos, wrote the draft of the manuscript and obtained the written consent. PR performed the literature review and participated in the manuscript writing. JG and WM helped to the final writing of the paper. All authors read and approved the final manuscript.

## Authors' information

MM is a Medical doctor and Lecturer in Anatomy and Cell Biology. PR is a Pathologist and Senior Lecturer in Pathology. JG is an ENT surgeon and a Senior Lecturer in Surgery. WM is a Professor of Cardiothoracic Surgery.
